# Incretins and cardiovascular disease: to the heart of type 2 diabetes?

**DOI:** 10.1007/s00125-023-05973-w

**Published:** 2023-08-05

**Authors:** Anna Solini, Domenico Tricò, Stefano Del Prato

**Affiliations:** 1 Department of Surgical, Medical, Molecular and Critical Area Pathology, University of Pisa, Pisa, Italy; 2 Department of Clinical and Experimental Medicine, University of Pisa, Pisa, Italy; 3 Interdisciplinary Research Center “Health Science”, Sant’Anna School of Advanced Studies, Pisa, Italy

**Keywords:** Cardiovascular diseases, Diabetes mellitus, type 2, Glucagon, Glucagon-like peptide-1, Glucose-dependent insulinotropic polypeptide, Heart failure, Incretins, Review

## Abstract

Major cardiovascular outcome trials and real-life observations have proven that glucagon-like peptide-1 (GLP-1) receptor agonists (GLP-1RAs), regardless of structural GLP-1 homology, exert clinically relevant cardiovascular protection. GLP-1RAs provide cardioprotective benefits through glycaemic and non-glycaemic effects, including improved insulin secretion and action, body-weight loss, blood-pressure lowering and improved lipid profile, as well as via direct effects on the heart and vasculature. These actions are likely combined with anti-inflammatory and antioxidant properties that translate into robust and consistent reductions in atherothrombotic events, particularly in people with type 2 diabetes and established atherosclerotic CVD. GLP-1RAs may also have an impact on obesity and chronic kidney disease, conditions for which cardiovascular risk-reducing options are limited. The available evidence has prompted professional and medical societies to recommend GLP-1RAs for mitigation of the cardiovascular risk in people with type 2 diabetes. This review summarises the clinical evidence for cardiovascular protection with use of GLP-1RAs and the main mechanisms underlying this effect. Moreover, it looks into how the availability of upcoming dual and triple incretin receptor agonists might expand the possibility for cardiovascular protection in people with type 2 diabetes.

**Graphical Abstract:**

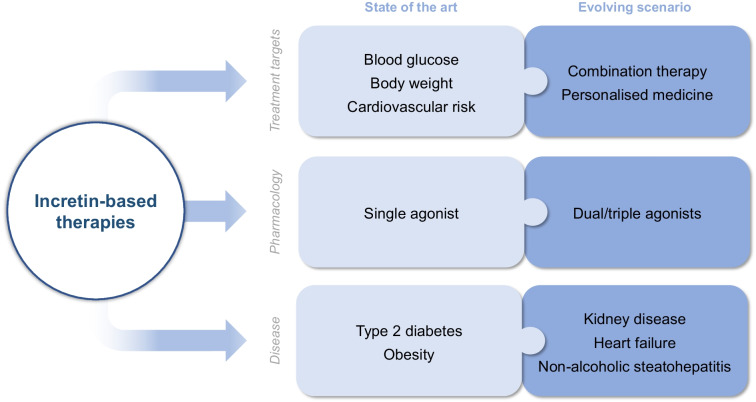

**Supplementary Information:**

The online version contains a slideset of the figures for download available at 10.1007/s00125-023-05973-w.


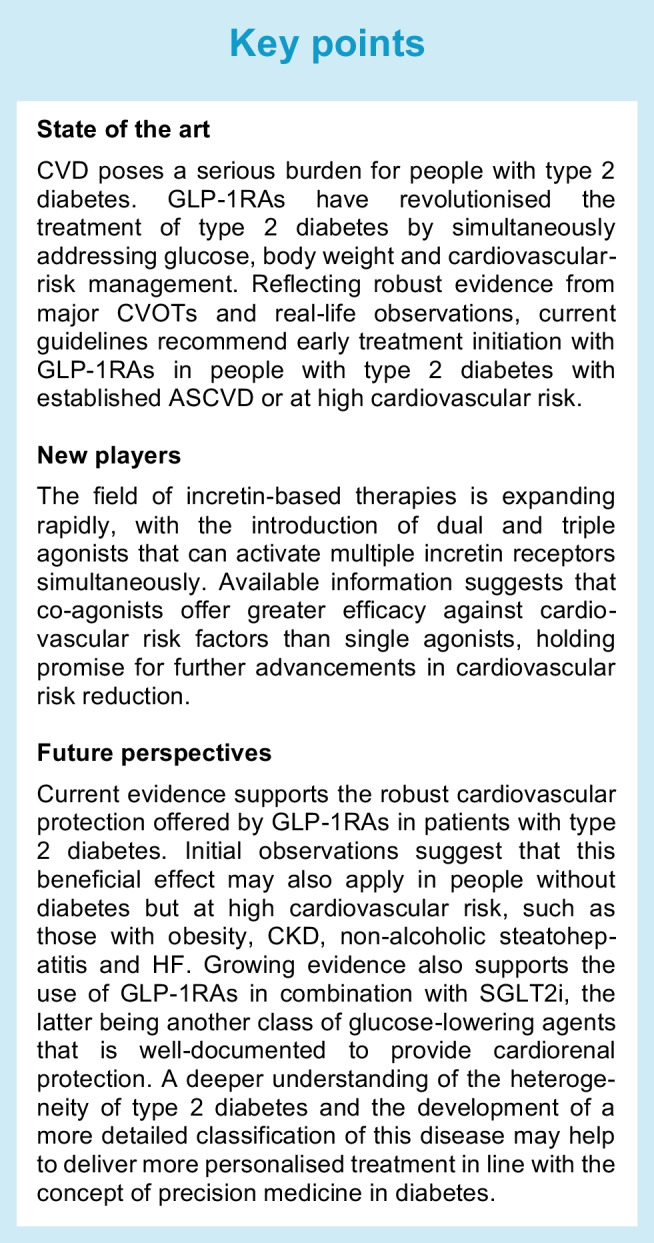


## The cardiovascular burden in type 2 diabetes

The goal of treatment in people with type 2 diabetes is delaying, if not preventing, the development of debilitating and sometime lethal complications. Among the latter, CVD still contributes to the excess of morbidity and mortality in type 2 diabetes [1]. As compared with individuals without diabetes, those with type 2 diabetes have at least a doubling of mortality rate irrespective of having or not having an established cardiovascular event [1]. For a long time, glycaemic control has represented the main goal of pharmacological treatment in type 2 diabetes. However, although good metabolic control can reduce the risk of microvascular complications [2], its impact on cardiovascular morbidity and mortality is still a matter of discussion. Compared with less-intensive glucose control, more intensive glucose control was indeed found to reduce the risk of major adverse cardiovascular events (MACEs) by 9% (HR 0.91 [95% CI 0.84, 0.99]) in previous trials largely based on metformin, sulfonylureas and insulin therapy [3]. This reduction in risk was primarily owing to a 15% reduced risk of myocardial infarction (HR 0.85 [95% CI 0.76, 0.94]) with no effect on cardiovascular mortality [3]. Starting in 2008, large cardiovascular outcome trials (CVOTs) have been carried out to test the safety and efficacy of new glucose-lowering agents, revealing the cardiovascular protective actions of glucagon-like peptide-1 (GLP-1) receptor agonists (GLP-1RAs). More recently, novel co-agonists of GLP-1, glucose-dependent insulinotropic polypeptide (GIP) and glucagon have also been approved or are under evaluation for the treatment of diabetes and associated metabolic diseases, holding potential for cardiovascular risk reduction. This review focuses on the cardiovascular protection associated with use of GLP-1RAs, and outlines how the field may evolve in the near future with the introduction of dual or triple incretin receptor co-agonists.

## Physiological actions of incretins in the heart and vasculature

**Fig. 1:**
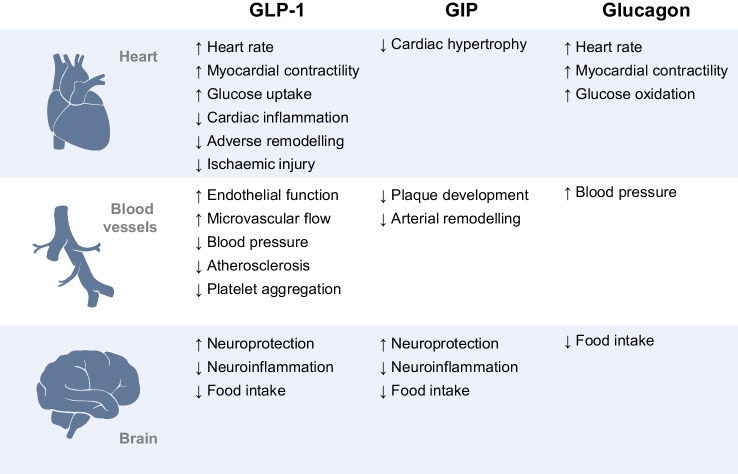
Proposed direct mechanisms of cardiovascular protection exerted by pharmacological activation of GLP-1, GIP and glucagon receptors. This figure is available as part of a downloadable slideset

The incretin family comprises peptides secreted in the gut that are derived from the proglucagon gene [4]. This family primarily includes GLP-1, GIP and glucagon. Although these peptides exert different metabolic effects, they share the ability to stimulate or potentiate insulin secretion in response to nutrient ingestion and reduce food intake. Several actions on the heart and vasculature have also been reported in experimental and clinical studies of incretin hormones and their analogues, as recently reviewed [4] (Fig. 1). These actions may be owing to direct activation of incretin hormone receptors, which are widely distributed in extra-pancreatic tissues, or mediated by indirect mechanisms. The results of CVOTs of GLP-1RAs support a positive effect of these agents on the cardiovascular system, although these results have been achieved using pharmacological levels of GLP-1 analogues. Of interest, no reduction in the risk for cardiovascular events was observed in CVOTs of dipeptidyl peptidase-4 inhibitors (DPP-4i), which prolong the half-life of endogenous GLP-1 and GIP [5–9]. The effect of GIP on the cardiovascular system is controversial, as much of the information in this area is largely based on experimental animal models, without a clear understanding of whether any of the observed activity also occurs in humans. In mice, inactivation of the GIP receptor was associated with improved outcomes after experimental myocardial infarction [10], whereas GIP infusion was found to exert significant anti-atherogenic effects in mouse models of diabetes [11]. GIP receptors have been reported in the human heart, and an epidemiological study in an elderly Swedish population observed an association between physiologically elevated levels of fasting GIP and increased intima–media thickness in the common carotid artery [12]. Furthermore, in a prospective, community-based study, elevated GIP levels were associated with greater risk of all-cause and cardiovascular mortality [13]. At a physiological concentration, glucagon is not believed to exert significant effects on the heart, while at supraphysiological concentrations it increases heart rate and cardiac contractility [14]. Because of these properties, glucagon has been evaluated for its potential to improve symptomatic heart failure (HF). However, most of these studies were of small size and lacking rigorous control and, as such, any cardioprotective effect of glucagon remains uncertain [15]. The available evidence for the cardiovascular effects of GIP has been reviewed in detail in this special issue [4, 16]. Also, while rigorous CVOTs have been carried out to assess safety and benefits of GLP-1RAs, no such studies are currently available for any other incretin. Therefore, we will focus on the cardiovascular effects of GLP-1RAs.

## The lessons from CVOTs

**Fig. 2:**
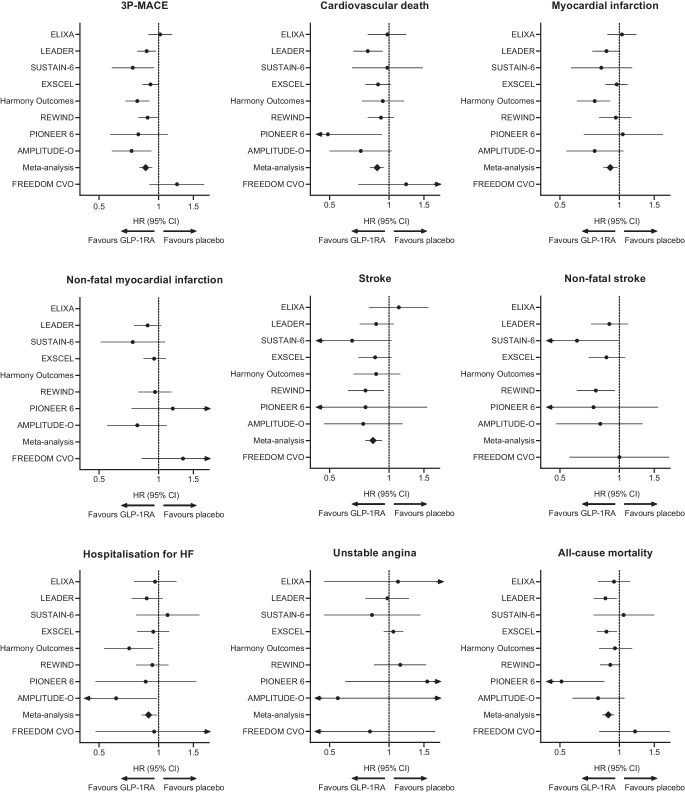
Cardiovascular outcomes and all-cause mortality for GLP-1RAs. Data were extracted from articles reporting primary results for the Evaluation of Lixisenatide in Acute Coronary Syndrome (ELIXA) [21], LEADER [17], SUSTAIN-6 [18], EXSCEL [23], Harmony Outcomes [19], REWIND [25], PIONEER 6 [22], AMPLITUDE-O [20] and FREEDOM CVO [24] trials, sorted by publication date, and from the meta-analysis by Sattar et al [26], which included all available data from GLP-1RA CVOTs except for FREEDOM CVO. 3P-MACE consisted of non-fatal myocardial infarction, non-fatal stroke and cardiovascular death. Data for myocardial infarction and stroke are for both fatal and non-fatal events. Data are plotted on a logarithmic scale (log_10_). This figure is available as part of a downloadable slideset

Since 2016, five dedicated CVOTs have demonstrated a significant reduction in the three-point MACE (3P-MACE) composite outcome, encompassing non-fatal myocardial infarction, non-fatal stroke and cardiovascular death, in patients with type 2 diabetes receiving long-acting GLP-1RAs (Fig. 2). In the Liraglutide Effect and Action in Diabetes: Evaluation of Cardiovascular Outcome Results (LEADER) [17] trial, the Trial to Evaluate Cardiovascular and Other Long-term Outcomes with Semaglutide in Subjects with Type 2 Diabetes (SUSTAIN-6) [18], the Harmony Outcomes [19] trial and the AMPLITUDE-O [20] trial, individuals with type 2 diabetes and at high risk of CVD (81–100% with established CVD) were randomly assigned to receive either GLP-1RA or placebo. During a median follow-up of 1.6–3.8 years, the relative risk of 3P-MACE was reduced by 13% in participants treated with once-daily liraglutide (HR 0.87 [95% CI 0.78, 0.97]) [17], and by 22–27% in participants treated with once-weekly injectable semaglutide (HR 0.74 [95% CI 0.58, 0.95]) [18], albiglutide (HR 0.78 [95% CI 0.68, 0.90]) [19] and efpeglenatide (HR 0.73 [95% CI 0.58, 0.92]) [20], compared with the placebo groups. Consistently, in the Researching Cardiovascular Events With a Weekly Incretin in Diabetes (REWIND) trial [21], treatment with dulaglutide reduced the rate of 3P-MACE by 12% (HR 0.88 [95% CI 0.79, 0.99]) in patients at lower CVD risk (31% with established CVD) during a longer follow-up period of 5.4 years. In contrast, four CVOTs reported neutral effects on 3P-MACE with long-term treatment with lixisenatide (the Evaluation of Lixisenatide in Acute Coronary Syndrome [ELIXA] trial) [21], oral semaglutide (the Peptide Innovation for Early Diabetes Treatment [PIONEER] 6 trial) [22], and exenatide prescribed as a once-weekly injection (Exenatide Study of Cardiovascular Event Lowering [EXSCEL]) [23] or administered via continuous subcutaneous release (FREEDOM Cardiovascular Outcomes [CVO]) [24] (Fig. 2). Discrepancies between CVOTs were also observed among individual 3P-MACE components: cardiovascular death was only found to be significantly reduced in the LEADER [17] and PIONEER 6 [22] trials; the rate of myocardial infarction (both fatal and non-fatal events) was significantly reduced in the LEADER [17] and Harmony Outcomes [19] trials; and the rate of stroke (combined fatal and non-fatal events) was significantly reduced in the REWIND trial only [25], while non-fatal stroke was reduced in the REWIND [25] and SUSTAIN-6 [18] trials (Fig. 2).

Heterogeneous populations and trial designs may account for some of these differences and preclude a direct comparison between benefits of different GLP-1RAs on morbidity and mortality; however, cardiovascular protection appears to be largely consistent across different members of this drug class (Fig. 2). In a meta-analysis of eight CVOTs, comprising 60,080 individuals, the relative risk of developing 3P-MACE with use of GLP-1RAs compared with placebo was reduced by 14% (HR 0.86 [95% CI 0.80, 0.93]) (Fig. 2), without statistical heterogeneity across subgroups stratified by trial duration, GLP-1RA dosing (daily vs weekly), human GLP-1 homology, participants’ age, BMI, baseline glycaemic control, cardiovascular risk and kidney function [26]. The inclusion of the FREEDOM CVO trial in the meta-analysis [24], which employed a subcutaneous delivery system to ensure long-lasting elevation of the circulating GLP-1 analogue, did not influence the benefits of GLP-1RAs on 3P-MACE (*n=*64,236; HR 0.87 [95% CI 0.81, 0.94]) [27]. Collectively, the greatest relative risk reduction was observed for stroke (−17%; HR 0.83 [95% CI 0.76, 0.92]), followed by cardiovascular death (−13%; HR 0.87 [95% CI 0.80, 0.94]), all-cause death (−12%; HR 0.88 [95% CI 0.82, 0.94]) and myocardial infarction (−10%; HR 0.90 [95% CI 0.83, 0.98]) (Fig. 2) [26].

In the same meta-analysis, the relative risk of hospital admission for HF was also reduced by 11% in patients treated with GLP-1RAs (HR 0.89 [95% CI 0.82, 0.98]) [26], confirming the beneficial effect previously observed only in the Harmony Outcomes [19] and AMPLITUDE-O [20] trials. These CVOTs reported the greatest numerical reductions in the rate of myocardial infarction, with the decrease being statistically significant in the Harmony Outcomes trial [19], raising the possibility that the benefit of GLP-1RAs on HF might reflect protective actions against myocardial damage. A recent meta-analysis corroborates this hypothesis, reporting that GLP-1RAs prevent hospitalisation for new-onset HF but do not reduce HF readmissions in patients with previous HF history [28].

Glycemia Reduction Approaches in Diabetes: A Comparative Effectiveness Study (GRADE) [29] has demonstrated that the cardiometabolic benefits of GLP-1RAs surpass that of older second-line glucose-lowering agents. In this RCT, 5047 patients with type 2 diabetes received either liraglutide, insulin glargine, glimepiride or sitagliptin in addition to metformin. In a prespecified secondary analysis, liraglutide reduced the relative risk for cardiovascular events by approximately 30% compared with sitagliptin (HR 0.68 [95% CI 0.51, 0.90]), glimepiride (HR 0.71 [95% CI 0.53, 0.93]) and the three other agents combined (HR 0.71 [95% CI 0.56, 0.90]) after a median follow-up of 5.0 years [29].

In summary, the available clinical evidence confirms that a significant cardiovascular protection is exerted by GLP-1RAs in type 2 diabetes patients who are at high cardiovascular risk or have established CVD. Given their body-weight lowering effect, it is also hypothesised that GLP-1RAs confer cardiovascular protection in obese individuals without diabetes but with established CVD. The ongoing Phase III Semaglutide Effects on Cardiovascular Outcomes in People With Overweight or Obesity (SELECT) trial (ClinicalTrials.gov registration no. NCT03574597) will be the first CVOT to evaluate superiority of a GLP-1RA (semaglutide 2.4 mg) vs placebo on 3P-MACE in obese individuals without diabetes. Moreover, the benefits of semaglutide in people with obesity-related HF with preserved ejection fraction (HFpEF), both with or without type 2 diabetes (ClinicalTrials.gov registration nos. NCT04916470 and NCT04788511, respectively), are under investigation.

## Real-world evidence of GLP-1RA effects on CVD

**Fig. 3:**
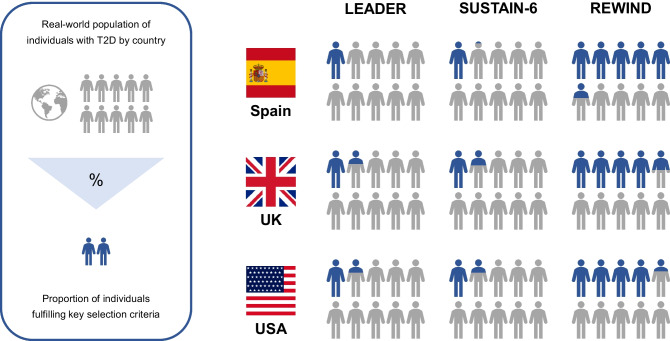
Generalisability of results from GLP-1RA CVOTs to the real-world type 2 diabetes population. The infographic shows the proportion of individuals in the general adult type 2 diabetes population from Spain [30], the UK [31], and the USA [32] that would have been eligible for enrolment in the LEADER [17], SUSTAIN-6 [18] and REWIND [25] trials (sorted by publication date) according to the trials’ key selection criteria. General population with type 2 diabetes, grey; eligible individuals for study enrolment, blue. T2D, type 2 diabetes. This figure is available as part of a downloadable slideset

Real-world observational studies performed in routine clinical settings are required to fully appreciate the potential for GLP-1RAs to elicit the cardiovascular protection that has been established in randomised clinical trials, using rigorous methodology. Because of such strict methodology, the populations included in CVOTs may not be representative of the general diabetes population. When the clinical characteristics of participants in the LEADER, REWIND and SUSTAIN-6 trials were compared with the typical diabetes population in Spain [30], the UK [31] and the USA [32], even the most representative of these CVOTs (REWIND) captured only a fraction (42.6–53.6%) of the real-world type 2 diabetes population (Fig. 3). Data from The Health Improvement Network (THIN) database, with contributions from over 640 UK general practices, have been used to compare matched type 2 diabetes patients who have been exposed (*n=*8345) or unexposed (*n=*16,541) to GLP-1RAs [33]. The primary outcome was all-cause mortality, while the secondary outcome was a composite of myocardial infarction and ischaemic heart disease, stroke and transitory ischaemic attack, and HF. Over a mean follow-up of 32 months, a total of 1146 deaths were recorded; individuals receiving a GLP-1RA were less likely to die from any cause compared with matched control participants (incidence rate ratio: 0.69 [95 %CI 0.61, 0.79]). A prospective observational study based on a Swedish registry (2010–2017) examined data from 17,868 people with type 2 diabetes who had survived their first myocardial infarction, 365 of whom were using GLP-1RAs (87% taking liraglutide, and 13% taking either exenatide, lixisenatide or dulaglutide) [34]. Over a 3 year follow-up, the use of GLP-1RAs was associated with a 28% reduction in the risk of MACE (adjusted HR 0.72 [95% CI 0.56, 0.92]) when compared with other glucose-lowering agents, primarily due to a lower rate of re-infarction and stroke. The use of GLP-1RAs was also associated with a 24% reduction in the risk of cardiovascular events in the first 30 days of follow-up (adjusted HR 0.76 [95% CI 0.58, 0.99]). Administrative claims data (2011–2015) from 11,351 type 2 diabetes patients who had previously been treated with metformin have recently been retrospectively analysed to compare CVD event rates following initiation of either liraglutide (66.3% of individuals), exenatide (29.6%) or other GLP-1RAs (4.1%) [35]. The primary outcome was time to first major CVD event (ischaemic heart disease, stroke, HF or peripheral arterial disease) after starting the GLP-1RA. Compared with liraglutide, initiating exenatide once weekly or twice daily early during the course of type 2 diabetes was not associated with a composite of major CVD events. The HR for an ischaemic event with exenatide once weekly relative to liraglutide was 1.85 (95% CI 0.97, 3.53). The analysis of another contemporary database from Slovenia, which included individuals with type 2 diabetes who were starting GLP-1RAs (*n=*855), DPP4i (*n=*3817) or sodium–glucose cotransporter 2 inhibitors (SGLT2i; *n=*2851) as add-on therapy to metformin (85% of individuals) and/or sulfonylureas (74% of individuals) showed that GLP-1RA combination therapy was associated with a lower risk of MACE (HR 0.64 [95% CI 0.43, 0.97]) and all-cause death (HR 0.53 [95% CI 0.35, 0.79]) compared with DPP4i, but not with SGLT2i. In contrast, no significant effect was observed with regard to CVD-related death or HF [36]. At odds with such findings, others have documented that SGLT2i exert stronger protection against MACE and HF vs GLP-1RAs [37], as previously observed in some RCTs.

Finally, one of the longest retrospective real-life observational studies to provide information on the relationship between the use of GLP-1RAs or SGLT2i and mortality comes from Italy [38]. In this study, death rates in 2010 and 2018 in the regions of Lombardy and Apulia were compared. Reductions in deaths were associated with the use of GLP-1RAs (HR 0.61 [95% CI 0.56, 0.65] and HR 0.63 [95% CI 0.55, 0.71], respectively), but these were numerically lower than the reductions obtained with SGLT2i (HR 0.47 [95% CI 0.40, 0.54] and HR 0.43 [95% CI 0.32, 0.57], respectively).

## Mechanisms of action of incretin-induced cardiovascular protection

Several direct and indirect mechanisms could account for the long-term cardioprotective actions of GLP-1RAs, which usually become evident 6–18 months after treatment initiation. These include the sustained reduction of multiple proatherogenic risk factors, encompassing hyperglycaemia, obesity, arterial hypertension, systemic and tissue inflammation, liver steatosis, dyslipidaemia, platelet aggregation and albuminuria [39]. Remarkable improvements in glycaemic control and body weight can certainly contribute to GLP-1RA-mediated cardiovascular benefits [40]. Mendelian randomisation studies support a causal role of genetically driven hyperglycaemia in atherosclerotic CVD (ASCVD) [41–43] and show that glucose-lowering variants of the GLP-1 receptor (GLP-1R) gene, *GLP1R*, confer protection against heart disease [44]. The glucoregulatory actions of GLP-1RAs are well known and include glucose-dependent stimulation of insulin secretion and inhibition of glucagon release, resulting in a marked reduction in HbA_1c_ in people with type 2 diabetes. By directly interacting with multiple hypothalamic circuits, GLP-1RAs also reduce appetite and food intake, leading to sustained weight loss in people with overweight and obesity [45]. Nevertheless, whether these mechanisms alone can fully explain the cardiovascular protection associated with GLP-1RA use has been questioned since investigators of the Harmony Outcomes trial found that the rate of MACEs decreased even in the absence of substantial HbA_1c_ and weight reductions during treatment with albiglutide [19].

The activation of extra-pancreatic GLP-1Rs may mediate direct benefits of GLP-1RAs on the heart and vasculature. Expression of GLP-1Rs has been detected at low levels in cardiomyocytes in atria and ventricles, endocardial and vascular endothelial cells, and vascular smooth muscle cells [46]. In clinical trials, GLP-1RAs modestly increased heart rate and reduced systolic blood pressure by up to 6 mmHg in hypertensive individuals [47], regardless of background blood-pressure-lowering therapy. Proposed mechanisms include increased natriuresis, vasorelaxation and secretion of cardiac natriuretic peptides [48], although these effects appear to be attenuated with chronic use of GLP-1RAs and have not been confirmed in all studies [49]. Clinical evidence also suggests that GLP-1RAs can enhance endothelium-dependent vasodilatation and microvascular recruitment [50], which may preserve ventricular function and survival under ischaemic conditions [46, 51–53].

Anti-inflammatory and antioxidant properties of GLP-1RAs, possibly mediated by GLP-1Rs expressed by gut intraepithelial lymphocytes and hepatic γδ T cells, have been reported in both animals [54] and humans [55]. Inflammatory cytokines and microbial infections enhance GLP-1 secretion by intestinal L cells, which, in turn, can attenuate colonic and systemic inflammation via GLP-1R signalling [56]. In people with non-alcoholic steatohepatitis (NASH), both liraglutide and semaglutide decreased liver inflammation and prevented fibrosis [57]. In mice, GLP-1RAs reduced aortic inflammation and prevented progression of atherosclerosis [58], whereas inconclusive findings have been reported in humans on the effects of GLP-1RA treatment on arterial inflammation [59] and carotid plaque composition and volume [60].

Treatment with GLP-1RAs rapidly reduces plasma triglyceride and LDL-cholesterol levels, even in the presence of statins [25]. GLP-1RAs also decrease postprandial triglyceride excursions by modulating gut chylomicron synthesis and secretion [61]. The improvements in fasting and postprandial dyslipidaemia are likely supported by concomitant body-weight loss and improved insulin sensitivity [62] given that GLP-1Rs are not expressed in the cells responsible for lipoprotein production, namely hepatocytes and enterocytes [63].

The regulation of platelet activation, thrombosis and platelet-mediated inflammation by GLP-1RAs is possibly mediated by GLP1Rs expressed on human platelets and represents another potential mechanism for reduced atherothrombotic events [64–66]. In fact, liraglutide attenuated thromboxane-induced platelet aggregation by 54% compared with sitagliptin or energy restriction in adults with impaired fasting glucose or impaired glucose tolerance [65], and a similar effect was observed in people with type 2 diabetes [66].

Accumulating evidence from clinical trials [67, 68] and real-world data [69] suggests that GLP-1RAs may offer additional renal benefits, which would translate into cardiovascular protection given the strong association between chronic kidney disease (CKD), ASCVD and HF [70]. While a dedicated kidney outcome trial is being conducted (the FLOW trial; ClinicalTrials.gov registration no. NCT03819153), secondary analyses of GLP-1RA CVOTs indicate that liraglutide [17], semaglutide [18], dulaglutide [25] and efpeglenatide [20] may reduce the relative risk of a composite kidney outcome, comprising new-onset macroalbuminuria, worsening kidney function, end-stage renal disease and renal death, by 15–36%. This effect is mainly driven by reduced macroalbuminuria, combined with a modest reduction in the slope of renal function decline [25, 67, 68].

Several mechanisms of protection against hospitalisation for HF have been demonstrated in different animal models, including increased myocardial glucose uptake, and reduced myocyte apoptosis, cardiac inflammation, adverse left ventricular remodelling, and atrial enlargement [71]. Nonetheless, these mechanisms have not been consistently replicated in humans, particularly in individuals with severely reduced left ventricular function who may not benefit [72, 73], or may even being harmed [74], from treatment with GLP-1RAs.

A clear explanation for the greater benefit of GLP-1RAs on stroke compared with myocardial infarction is still lacking [26]. GLP-1RAs neither reduce the risks for atrial fibrillation (and, thus, cerebral embolism) [75] or haemorrhagic stroke [76], nor do they acutely affect cerebral blood flow velocity [77]. However, direct anti-inflammatory, antioxidant, neurotrophic and neuroprotective actions of GLP-1RAs and GIP/GLP-1 co-agonists have been reported in animal models of stroke and neurodegenerative diseases [78].

## An evolving scenario

The results from CVOTs have set an unprecedented scenario and have provided a step forward to reducing cardiovascular burden in people with type 2 diabetes. Use of higher doses of dulaglutide [79] and semaglutide [80] than those initially approved for the treatment of type 2 diabetes have been shown to offer an even larger effect in terms of glycaemic control and body-weight reduction. However, whether this may also translate into greater cardiovascular protection has not been assessed. Furthermore, the advancement of precision medicine approaches, which combine progress in pharmacogenomics with established knowledge of the heterogeneous pathological processes underlying the aetiology of type 2 diabetes, holds the potential to uncover distinct subsets of individuals who are differentially responsive to the beneficial cardiometabolic actions of incretin-based therapies [81]. This novel approach would explain the varying responses to GLP-1RAs observed in clinical trials and may help to facilitate clinical decision-making, allowing for individualised treatment selection and optimised outcomes. As mentioned, along with GLP-1RAs, evidence has been gathered for the cardiovascular protective actions of SGLT2i, with the latter conferring a more pronounced protection with respect to HF and CKD, whereas GLP-1RAs may be associated with a greater benefit in relation to fatal and non-fatal stroke [82]. Current guidelines reflect this compelling evidence and indicate early treatment initiation with GLP-1RAs and/or SGLT2i in the primary and secondary prevention of cardiovascular and renal events in people with type 2 diabetes [83]. Of interest, the mechanisms of action of these two classes of drugs are different and potentially complementary, supporting the combined use of GLP-1RAs and SGLT2i in people with type 2 diabetes [84–89]. In the EXSCEL CVOT trial, 8.7% of participants randomized to exenatide once weekly were also started on an SGLT2i. Upon propensity matching, the risk for all-cause mortality with the combined use of exenatide and SGLT2i was significantly lower compared with placebo (HR 0.38 [95% CI 0.16, 0.90]) and exenatide alone (HR 0.41 [95% CI 0.17, 0.95]) [84]. In an exploratory analysis of the AMPLITUDE-O trial, which had the highest proportion (15.2%) of participants receiving SGLT2i at baseline among the available GLP-1RA CVOTs [20], the efficacy of efpeglenatide on preventing 3P-MACE and associated cardiometabolic variables appeared to be independent of concurrent SGLT2i use [85].

Previous work has highlighted potential synergistic effects among members of the incretin family, leading to the development of multi-incretin agonists [90]. Among those in a more advanced stage of development are the GLP-1/glucagon and in particular the GIP/GLP-1 dual agonists, of which tirzepatide was the first to be made available in the USA and is expected to enter Europe soon. The use of this GIP/GLP-1 dual agonist in individuals with type 2 diabetes has shown superiority with respect to HbA_1c_ lowering, body-weight reduction, improvement in lipid profile and reduction of arterial blood pressure, as compared with placebo, semaglutide or insulin [91, 92]. Moreover, post hoc analyses have provided initial support to an effect in terms of renal protection [93] and reductions in liver steatosis [94], two conditions contributing to cardiovascular risk in diabetes. In a meta-analysis including data from 4887 participants treated with tirzepatide and 2328 control participants, an HR of 0.80 (95% CI 0.57, 1.11) was calculated for four-point MACE (MACE-4), 0.90 (95% CI 0.50, 1.61) for cardiovascular death and 0.80 (95% CI 0.51, 1.25) for all-cause mortality [95]. However, the results of the SURPASS-CVOT trial (ClinicalTrials.gov registration no. NCT04255433) will better define the cardiovascular safety and benefit of tirzepatide as compared with dulaglutide in type 2 diabetes patients with established ASCVD.

The next chapter of incretin-based therapy for lowering cardiovascular risk in type 2 diabetes will be represented, most likely, by the development of tri-agonists capable of simultaneous activation of GLP-1, GIP and glucagon receptors. Encouraging results of a Phase Ib trial with retatrutide, an incretin tri-agonist, have shown an acceptable safety profile, suitability for once-weekly dosing, and robust reductions in glucose levels and body weight [96].

## Conclusion

The incretin concept was initially developed to account for gut factors able to stimulate and potentiate insulin secretion [4]. The first peptide to attract attention was GIP, soon followed by GLP-1 because of its more suitable features as a pharmacological agent for improvement of glucose control and body-weight reduction in people with type 2 diabetes [4]. Glucagon has always been considered a main player in diabetes, as a counterregulatory hormone, although some interest was transiently generated because of its inotropic and chronotropic action [16]. However, it was only with the results of CVOTs based on GLP-1RAs that a new era in the treatment of type 2 diabetes has been triggered: together with SGLT2i, for the first time, it has been possible to go to the heart of the problem of type 2 diabetes, i.e. its cardiovascular complications. These results have revolutionised the therapeutic management of diabetes, moving from mere glycaemic targets towards a more holistic approach to the disease [83]. GLP-1RAs, for the first time, have permitted simultaneous control of glucose, body weight and cardiovascular risk factors, and enabled us to confer effective cardioprotection in those with organ damage. Despite the incretin chapter opening more than 100 years ago [4], it is now moving with full steam ahead by taking advantage of simultaneous multi-incretin agonism. We are now at the heart of the problem and will look for even more efficient ways to prevent CVD in a growing number of people with (and possibly without) diabetes.

## Supplementary Information

Below is the link to the electronic supplementary material.

Supplementary file1 (431 KB)

## Abbreviations

ASCVD: Atherosclerotic CVD
CKD: Chronic kidney disease
CVOT: Cardiovascular outcome trial
DPP-4i: Dipeptidyl peptidase-4 inhibitors
EXSCEL: Exenatide Study of Cardiovascular Event Lowering
FREEDOM CVO: FREEDOM Cardiovascular Outcomes
GIP: Glucose-dependent insulinotropic polypeptide
GLP-1: Glucagon-like peptide-1
GLP-1R: Glucagon-like peptide-1 receptor
GLP-1RA: Glucagon-like peptide-1 receptor agonist
HF: Heart failure
LEADER: Liraglutide Effect and Action in Diabetes: Evaluation of Cardiovascular Outcome Results
MACE: Major adverse cardiovascular event
PIONEER: Peptide Innovation for Early Diabetes Treatment
3P-MACE: Three-point major adverse cardiovascular event
REWIND: Researching Cardiovascular Events With a Weekly Incretin in Diabetes
SGLT2i: Sodium–glucose cotransporter 2 inhibitors
SUSTAIN-6: Trial to Evaluate Cardiovascular and Other Long-term Outcomes with Semaglutide in Subjects with Type 2 Diabetes

## Authors’ relationships and activities

AS has received research grants to the institution from Sankyo and Sanofi; has served as an advisor for Boehringer Ingelheim, Eli Lilly, Novo Nordisk, and Sanofi; and has received fees for speaking from Sanofi, Bayer, Boehringer Ingelheim, Novo Nordisk and Sankyo. DT has served as an advisor for Amarin; and has received fees for speaking from AstraZeneca, Eli Lilly & Co. and Novo Nordisk. SDP has served as president of EASD/European Foundation for the Study of Diabetes (EFSD) (2020–2022); has received research grants to the institution from AstraZeneca and Boehringer Ingelheim; has served as advisor for Abbott, Applied Therapeutics, AstraZeneca, Boehringer Ingelheim, Eli Lilly & Co., EvaPharma, Jiangsu Hengrui Pharmaceuticals Co., Menarini International, Merck Sharpe & Dohme, Novartis Pharmaceutical Co., Novo Nordisk, Sanofi and Sun Pharmaceuticals; and has received fees for speaking from AstraZeneca, Boehringer Ingelheim, Eli Lilly & Co., Laboratori Guidotti, Merck Sharpe & Dohme and Novo Nordisk. The authors declare that there are no other relationships or activities that might bias, or be perceived to bias, their work.
