# Supplementary material for: Incretins and cardiovascular disease: to the heart of type 2 diabetes?
Source: Diabetologia. 2023 Aug 5;66(10):1820–31. doi: 10.1007/s00125-023-05973-w (PMC10473999; doi:10.1007/s00125-023-05973-w)
Supplement: Supplementary file 1 — Supplementary file1 (431 KB) [file 125_2023_5973_MOESM1_ESM.pptx]

## Slide 1
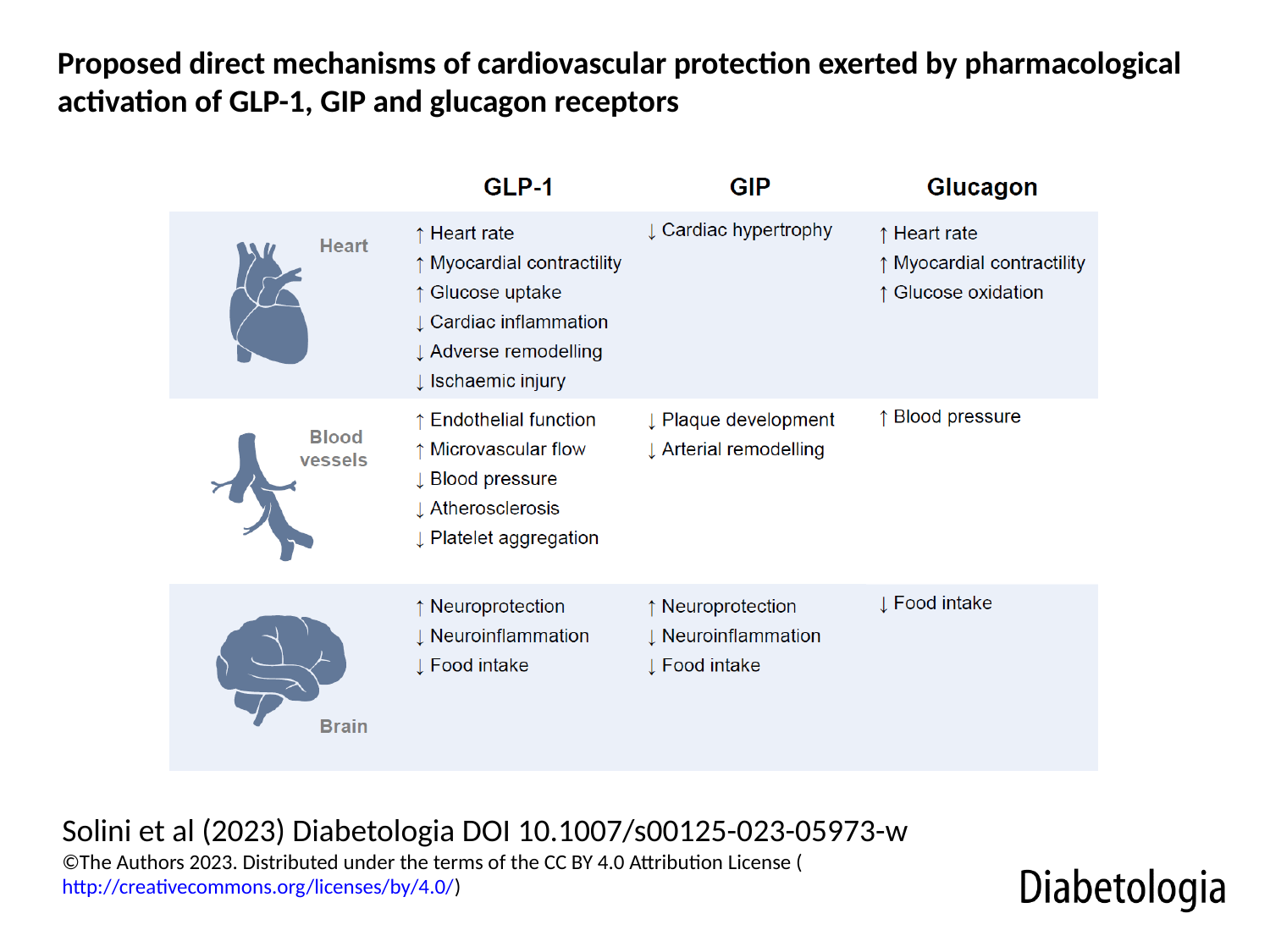

Proposed direct mechanisms of cardiovascular protection exerted by pharmacological activation of GLP-1, GIP and glucagon receptors
Solini et al (2023) Diabetologia DOI 10.1007/s00125-023-05973-w
©The Authors 2023. Distributed under the terms of the CC BY 4.0 Attribution License (http://creativecommons.org/licenses/by/4.0/)

## Slide 2
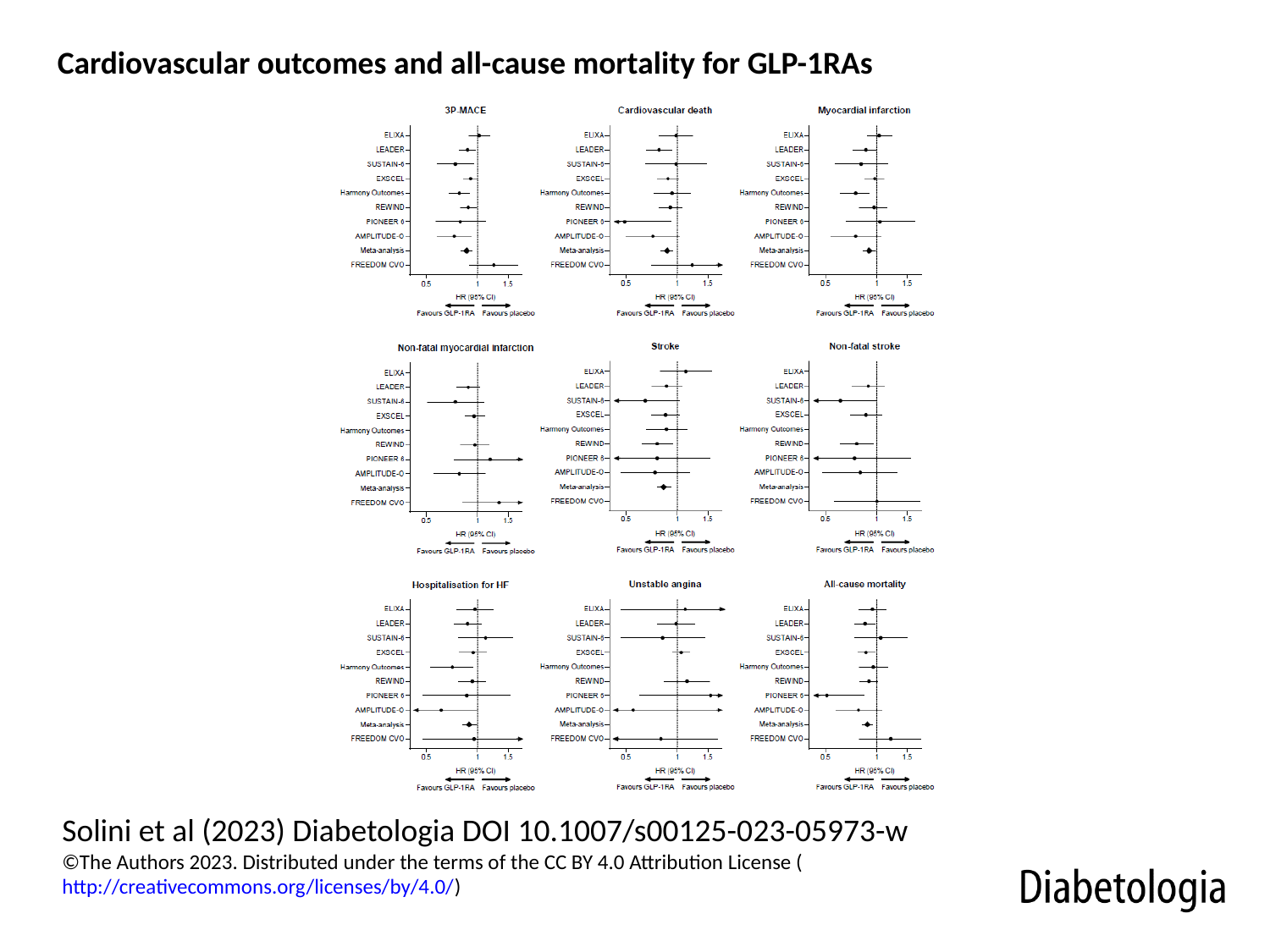

Cardiovascular outcomes and all-cause mortality for GLP-1RAs
Solini et al (2023) Diabetologia DOI 10.1007/s00125-023-05973-w
©The Authors 2023. Distributed under the terms of the CC BY 4.0 Attribution License (http://creativecommons.org/licenses/by/4.0/)

## Slide 3
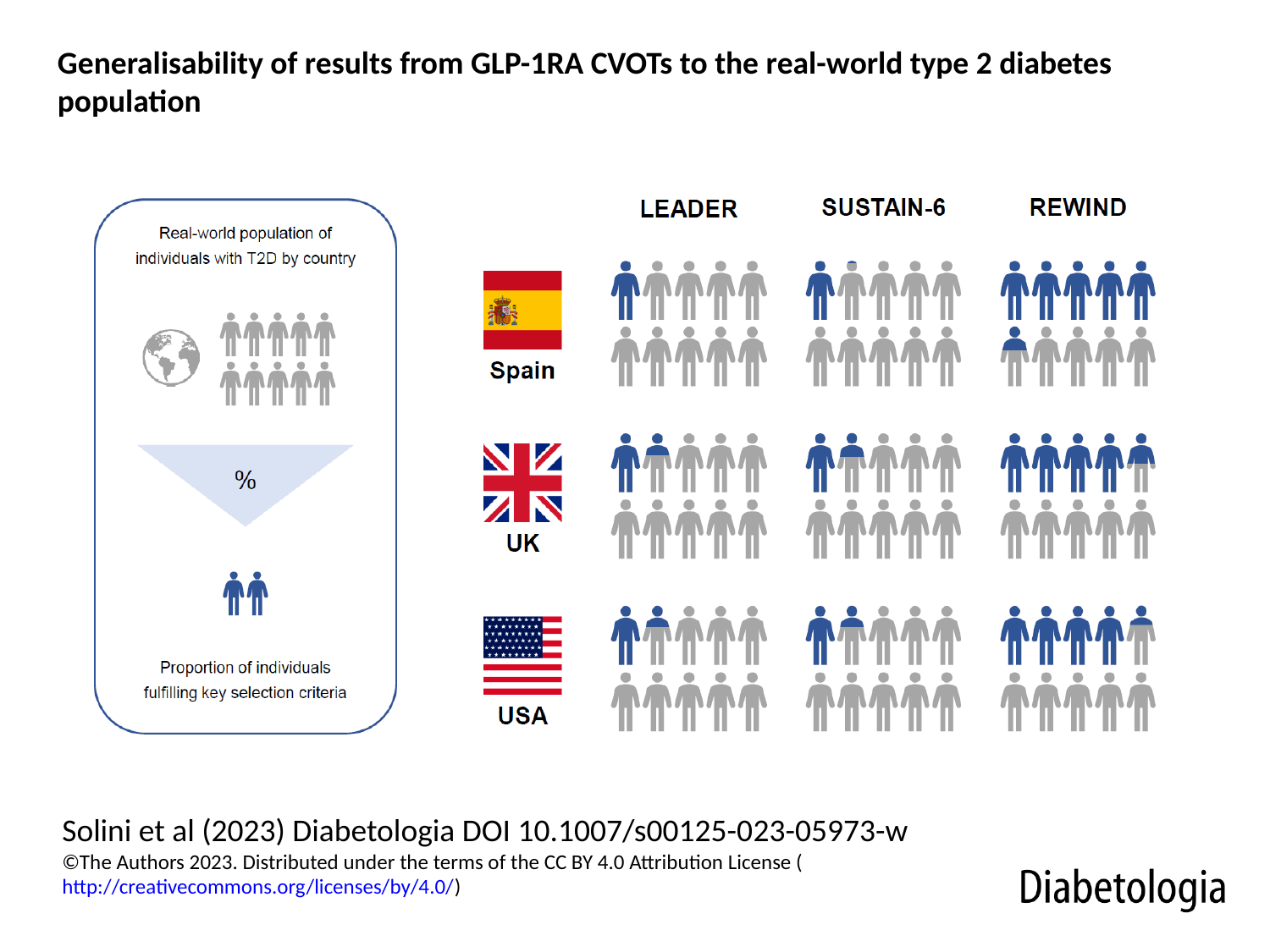

Generalisability of results from GLP-1RA CVOTs to the real-world type 2 diabetes population
Solini et al (2023) Diabetologia DOI 10.1007/s00125-023-05973-w
©The Authors 2023. Distributed under the terms of the CC BY 4.0 Attribution License (http://creativecommons.org/licenses/by/4.0/)
